# Implementation of the International Health Regulations (2005) Through Cooperative Bioengagement

**DOI:** 10.3389/fpubh.2015.00231

**Published:** 2015-10-13

**Authors:** Claire J. Standley, Erin M. Sorrell, Sarah Kornblet, Julie E. Fischer, Rebecca Katz

**Affiliations:** ^1^Global Health Security Program, Department of Health Policy and Management, Milken Institute School of Public Health, The George Washington University, Washington, DC, USA

**Keywords:** International Health Regulations, Global Health Security Agenda, biological threat reduction, cooperative bioengagement, health systems strengthening

## Abstract

Cooperative bioengagement efforts, as practiced by U.S. government-funded entities, such as the Defense Threat Reduction Agency’s Cooperative Biological Engagement Program, the State Department’s Biosecurity Engagement Program, and parallel programs in other countries, exist at the nexus between public health and security. These programs have an explicit emphasis on developing projects that address the priorities of the partner country as well as the donor. While the objectives of cooperative bioengagement programs focus on reducing the potential for accidental or intentional misuse and/or release of dangerous biological agents, many partner countries are interested in bioengagement as a means to improve basic public health capacities. This article examines the extent to which cooperative bioengagement projects address public health capacity building under the revised International Health Regulations and alignment with the Global Health Security Agenda action packages.

## Introduction

The concept of “cooperative threat reduction” (CTR) was introduced in the years immediately following the collapse of the former Soviet Union (FSU), when concerns abounded that the equipment, expertise, and materials used as part of state-supported nuclear programs were suddenly vulnerable to exploitation and misuse (1). To counter this apparent threat, Senators Nunn and Luger spearheaded the passage of legislation to form US government programs that would engage the newly formed nations of the FSU in rebuilding peaceful, civilian research, and development capabilities while also reducing the threat that nefarious state or non-state actors would gain access to capabilities for producing an unconventional weapon. From the first Department of Defense programs to assist in safely dismantling the Soviet nuclear arsenal authorized by The Soviet Nuclear Threat Reduction Act of 1991 (commonly known as the Nunn-Lugar Act) (2), CTR programs expanded to encompass collaborative efforts by the Departments of Defense, State, and Energy to secure nuclear, chemical, and biological threats throughout the FSU. After the fall of Saddam Hussein’s regime in 2003, the State Department recognized the similar need to engage Iraqi scientists who had previously been employed in state-run weapons programs, and thus CTR programs expanded beyond the FSU. CTR has become a global enterprise, with more than $1 billion in US funds annually supporting partnerships in countries on every continent (3, 4).

A hallmark of modern CTR programs is the recognized need to develop projects that meet partner country needs and priorities as well as the goal of reduced threat of misuse or proliferation (5, 6). In the last decade, the CTR framework with respect to biological agents shifted from “cooperative biological threat reduction” to “cooperative biological engagement,” or bioengagement, reflecting the transition away from destruction of biological munitions and related manufacturing capabilities to an emphasis on prevention. There are also some key distinctions from parallel chemical and radio-nuclear programs. First, most high-priority biological agents occur naturally in the environment. These pathogens cause disease outbreaks that can severely affect public health and development, and economic stability, and may be difficult to distinguish from an intentional attack without further investigation. Cooperative bioengagement efforts to date have focused on prevention of acquisition from the environment, along with developing enhanced security and safety at laboratories, an emphasis on responsible conduct of research, and improved capacities to detect disease outbreaks and other unusual events (7, 8). These areas of focus aim at limiting the opportunities for accidental or intentional release of a pathogen as well as improving the likelihood that the US and the broader international community would be alerted quickly of any suspicious outbreak. Because many of the biological agents deemed high risk for weaponization by the US and others are also high-priority endemic or epidemic-prone diseases of public health significance, cooperative bioengagement can provide a mutually acceptable platform for forming new country partnerships, particularly in vulnerable or insecure regions or countries where a traditional “security” program might be politically sensitive or operationally limited.

Cooperative bioengagement allows projects to be developed that meet both public health and security objectives, providing incentives for partner countries who often have health and development concerns foremost in their national priorities. These priorities may even be driven by international legal obligations. For example, the World Health Organization’s revised International Health Regulations (IHR) mandate that all 196 States Parties develop the core capacities needed to detect, assess, report, and respond to events that could constitute a public health emergency of international concern (PHEIC). By June 2014, the end date of the first 2-year extension for implementation, only 64 countries out of the 196 States Parties to the IHR declared that they had met these minimum core capacity requirements (9). Those States Parties that are not yet in compliance have until June 2016 to develop the necessary capacities, indicating significant opportunity for partnership with the international community, provided objectives of both sides are aligned.

Recognizing the potential benefits of multi-sectoral collaboration with respect to controlling disease outbreaks, in February 2014, 29 countries, together with WHO, the Food and Agriculture Organization (FAO) and the World Organisation for Animal Health (OIE) announced the launch of the Global Health Security Agenda (GHSA). While not binding, the GHSA represents a partnership of now over 40 countries committed to accelerating and elevating progress toward “a world safe and secure from infectious disease threats” (10).

In this paper, we sought to identify existing efforts on the complementarity between health security frameworks (11, 12), and explore the extent to which IHR and GHSA overlap with priorities for developing and executing bioengagement programs. Given cooperative bioengagement’s emphasis on country partnership and interest in human and animal pathogens, we hypothesized at least some alignment with IHR and GHSA; indeed, through a descriptive mapping exercise and a series of case studies, we demonstrate that cooperative bioengagement provides significant, although imperfect, alignment with these existing health security frameworks.

## Materials and Methods

Using open source material, we mapped cooperative bioengagement priorities, based on four examples of bioengagement programs, against the core capacity indicators for the IHR and GHSA action packages. We then identified three projects, funded by bioengagement programs, to use as case studies to examine how these efforts aligned with public health capacity building priorities, opportunities for greater cooperative programing, and the potential challenges to achieving cooperative bioengagement aims through the lens of IHR and GHSA.

### Cooperative Bioengagement Priorities

Using open source material, we first sought to characterize common elements across different biological engagement programs. Programs were selected using the following criteria:

• Information on objectives and program mission were available online
• The program is driven by a security and/or non-proliferation mandate.

Using these criteria, we identified the following four programs to analyze for common themes:

• U.S. Defense Threat Reduction Agency Cooperative Biological Engagement Program (CBEP)
• U.S. Department of State Biosecurity Engagement Program (US BEP)
• United Kingdom Biological Engagement Program (UK BEP)
• Canadian Global Partnership Program (GPP).

As a framework for identifying common mission elements between these programs, we used the three “pillars” identified by CBEP as categories for their programmatic activities, which are “biosafety and biosecurity capacity building,” “disease surveillance, detection, diagnosis, and control” (sometimes referred to as “biosurveillance”), and “cooperative biological research” (7, 13).

### Mapping Health Security Frameworks Against Bioengagement Pillars

The IHR (14) are a legally binding agreement on health security issues for all Member States of the World Health Assembly, and a key framework against which to map bioengagement priorities. In addition, given its high political profile in the health security community since its launch in 2014, we also selected the GHSA, a framework intended to promote accelerated implementation of IHR, and other supporting health security frameworks for analysis.

**Figure 1:**
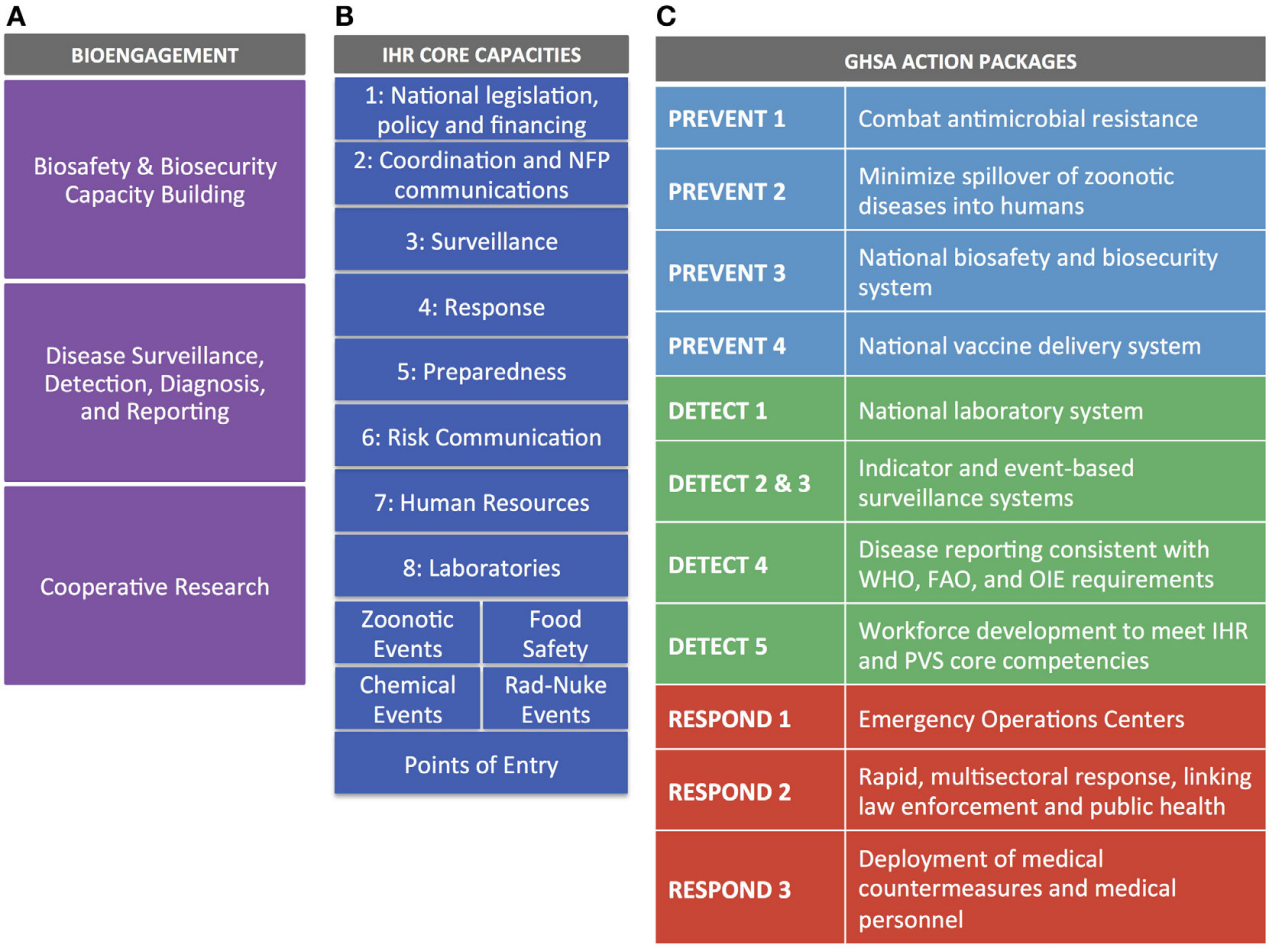
**Visual representation of the bioengagement pillars (A); IHR core capacities (B); and Global Health Security Agenda (GHSA) action packages (C) included in the mapping exercise**.

For IHR, we mapped each of the eight core capacities, the four specific hazards, and Points of Entry against cooperative bioengagement priorities, assessing the content of each based on the indicators and attributes contained within the IHR Core Capacity Monitoring Framework (2013) (15). For GHSA, we examined each of the 11 action packages, and their corresponding targets and measures (16), to identify elements to map back to the three bioengagement pillars. Figure 1 demonstrates the elements of bioengagement, IHR, and GHSA that were used in the mapping process. We arrayed all of these elements in a matrix to facilitate comparison.

### Case Studies in Bioengagement

To demonstrate how the elements of IHR and GHSA map to the bioengagement priorities, we examined three case studies. We selected these case studies to be descriptive illustrations of bioengagement programs, and thus was a convenience sample representing each of the three pillars, and with sufficient publicly available information to identify project focus areas and goals. We used open source and online material to describe the following bioengagement projects:

• Development of Uganda’s Biosecurity Policy and Bill
• Launch of the Republic of Kenya’s Zoonotic Disease Unit (ZDU)
• Iraq Science Fellowship Program (ISFP).

For each case study, stated project objectives were categorized per three bioengagement pillars. The project objectives were also examined in terms of their alignment with IHR core capacities and GHSA action packages.

## Results

### Bioengagement Priorities

The programmatic focus areas, for each of the four bioengagement programs analyzed (CBEP, US BEP, UK BEP, and GPP), were generally well characterized by the three proposed pillars of “biosafety and biosecurity capacity building,” “disease surveillance, detection, diagnosis, and control,” and “cooperative biological research.” Elements that were not well captured by these categories but were expressed as objectives of the four bioengagement programs included an emphasis on engagement and programmatics in certain countries and regions [including explicit geographic prioritization by “threat” although the criteria for determining level of threat are not always clearly defined (17)]; a focus on adherence to global standards and norms, including the Biological and Toxins Weapons Convention (BWC) (18, 19); and interest in projects relating to bioethics or addressing dual-use research of concern (DURC) (18). Table 1 outlines the main programmatic focus areas we identified for each program, categorized by bioengagement pillar.

**Table 1:** **Bioengagement programmatic efforts and categorization into “pillars” corresponding to “Biological Safety and Security,” “disease surveillance, detection, diagnosis, and reporting,” and “cooperative biological research**.”

Program	Biological safety and security	Disease surveillance, detection, diagnosis, and reporting	Cooperative biological research	Other stated priorities?
US CBEP (13, 34)	Consolidation and security of dangerous pathogen collections	Improved capabilities to detect, diagnose, and report outbreaks	Engage scientists in health security research	
	Safety and security of biological facilities		Collaborative research to detect biothreats	
US BEP (17, 35, 36)	Risk assessment	Improved detection and control of priority diseases	Scientist engagement	Explicitly prioritized by threat
	Laboratory security upgrades	Field epidemiology training	Joint scientific collaborations	Reinforce global norms (i.e., BWC)
	Biorisk management training	Surveillance for priority diseases	Research that advances health security	Sustainability
	Biosafety associations		Training and research grants	
	“Holistic” biosecurity (i.e., law enforcement)			
GPP (19, 37)	New lab and facility upgrades	New lab and facility upgrades	Scientist redirection	Guidelines and standards
	Biosafety associations	Diagnostic training		Non-proliferation initiatives
	Biosafety/security training			
UK BEP (18, 38, 39)	Safety and security training packages	Molecular diagnostics training	Redirection of former weapons scientists	BTWC awareness and implementation
	Biosafety associations	Laboratory capacity building	Collaborative research	Dual use and bioethics training
	Physical security and inventory			

*US CBEP, U.S. Defense Threat Reduction Program Cooperative Biological Engagement Program; US BEP, U.S. Department of State Biosecurity Engagement Program; GPP, Canadian Department of Foreign Affairs, Trade, and Development Global Partnership Program; UK BEP, United Kingdom Ministry of Defense Biological Engagement Program*.

### Bioengagement Priorities and Alignment with IHR and GHSA

**Figure 2:**
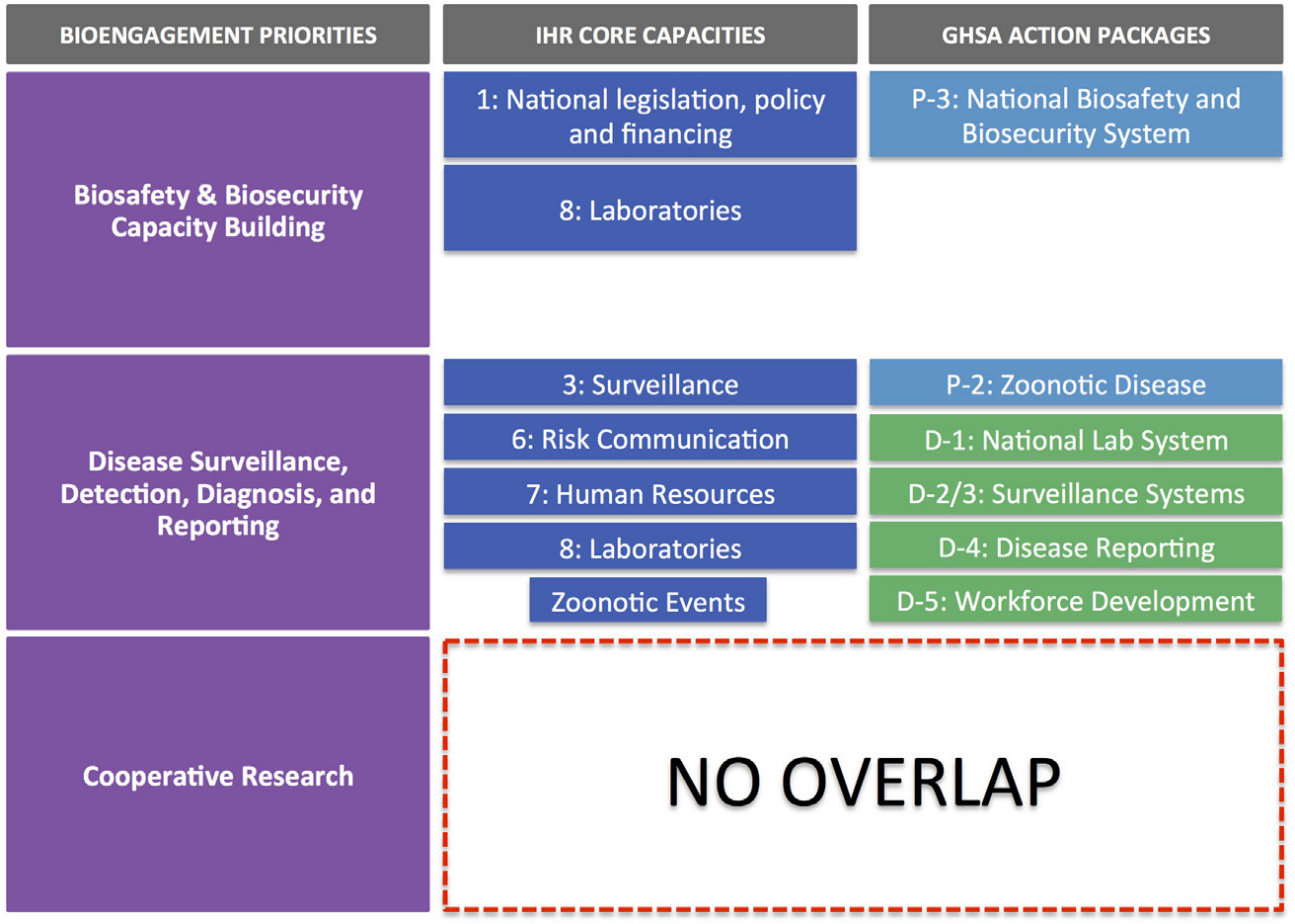
**IHR core capacities and GHSA action packages mapped against the three pillars of bioengagement programs**. Note that there is no overlap of IHR and GHSA core capacities and action packages with the “cooperative biological research” pillar of bioengagement.

Figure 2 illustrates the alignment of IHR core capacity attributes and GHSA action package targets, organized against the pillars of bioengagement. Through this exercise, we came away with three major findings:

(1) Extensive overlap of priorities exists with respect to capacity building for disease surveillance, detection, diagnosis, and control. These capabilities are a priority across both IHR and GHSA frameworks as well as a stated priority for all four bioengagement programs that we included in our analysis. However, it is important to note that most of the bioengagement programs qualify support for this focus area by noting that projects must focus on “priority” pathogens. Similarly, under the “Detect” action packages, GHSA measures the ability to perform surveillance and appropriate diagnostic tests for a limited number of priority syndromes and diseases, while allowing flexibility in determining those priorities. IHR’s emphasis is solely on events that could constitute a public health event of international concern, so while certain epidemic-prone diseases are highlighted in Annex 2 (14) as always requiring notification to WHO (or at least requiring critical evaluation of whether notification is necessary), the core capacities needed to identify such events must, by definition, be capable of detecting and reporting all outbreaks.
(2) Significant overlap exists between IHR, GHSA, and bioengagement under the biosafety and biosecurity capacity building pillar, but the range of activities varies. Within the IHR Monitoring Framework, Core Capacities 1 (National legislation, policy, and financing) and eight (Laboratories) address separate elements of this pillar: the ability to develop and implement legislation or regulations and laboratory biosafety and biosecurity, respectively. This latter indicator also includes policies or regulations required above the institutional level. However, IHR does not emphasize the importance of inventory and physical security of pathogens, readily provide a forum for engaging biosafety associations, nor explicitly cover non-diagnostic/clinical settings. GHSA’s Prevent 3 action package (biosafety and biosecurity) is more comprehensive, covering all facilities handling especially dangerous pathogens and including pathogen security as a key part of the target (but not a measure of implementation success). While implied, Prevent 3 lacks an explicit mention of using a risk assessment-driven approach to biosafety and biosecurity, which is mentioned as a priority under several bioengagement programs. Under Respond 2 (Linking Public Health with Law Enforcement), GHSA touches on concepts related to “holistic” biosecurity stated in US BEP’s program priorities (17) though the bioengagement emphasis is on creating public health and law enforcement linkages to prevent an attack, whereas GHSA is more focused on the response element.
(3) Cooperative biological research is a pillar that does not present any clear alignment with the IHR core capacity indicators nor the GHSA action package targets and measures. As such, our analysis indicated that as defined by most programs, cooperative biological research would not provide a means for partner countries to achieve any aspects of IHR or GHSA compliance. This does not preclude activities that do further IHR and GHSA aims from simultaneously engaging biological scientists in a similar fashion to that achieved by designated cooperative biological research projects; however, the form of the engagement and the way the project is developed are likely to be significantly different from a traditional research project, where the emphasis is on hypothesis-driven investigation, rather than relationship building, training, or applicability to public health.

### Cooperative Bioengagement in Action: Case Studies

In order to further explore the extent of overlap between bioengagement priorities and the IHR/GHSA frameworks, we selected three case studies, each representing one of the three main pillars of bioengagement. Through this process, we were able to confirm some of the observations from the *a priori* mapping exercise, while also identify other observations related to the opportunities and challenges of aligning bioengagement programs with implementation of health security frameworks.

#### Uganda Biosecurity Policy and Bill

In 2012, the Uganda National Council for Science & Technology (UNCST), in collaboration with Global Implementation Solutions (GIS) and with funding support from the US BEP, held a consultative workshop to discuss the development of a new national biosecurity policy (20, 21). The intention was to create a biosecurity policy that would build on and complement Uganda’s existing 2008 biotechnology and biosafety policy, and specifically address biosecurity issues as well as Uganda’s obligations under the BWC. Since the initial kick-off meeting, there have been a number of additional consultations and sensitization efforts, involving broad representation from Ugandan government ministries as well as a variety of other stakeholders. A Bill, written based on the policy, will be submitted to the Ugandan Parliament by the end of 2015 (21).

**Figure 3:**
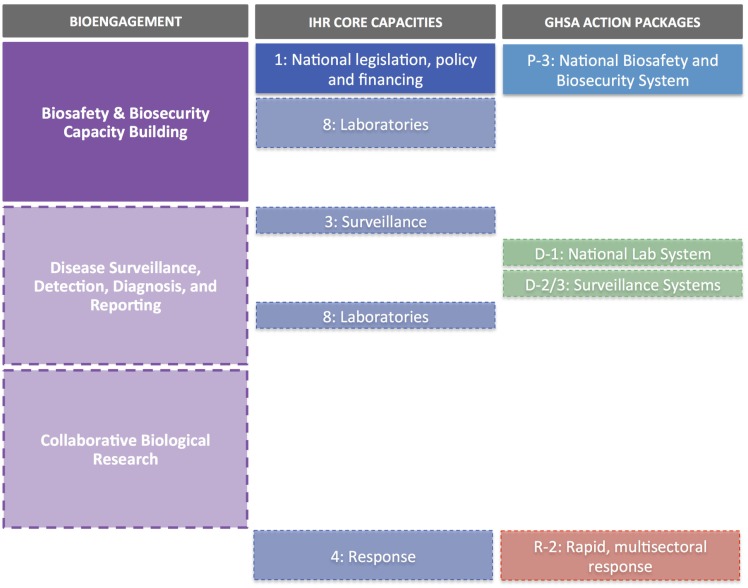
**Uganda Biosecurity Policy and Bill case study**. Solid boxes indicate elements supported directly by the development of the Policy and Bill; transparent boxes indicate additional elements which would be addressed through implementation of the Bill (and after approval by the Ugandan Parliament).

Based on stated objectives from UNCST, the Bill will cover seven main objectives (Table 2). These outcomes span preparedness, early detection of disease threats, and integrated response to emerging events as well as a recognition of the importance of collaborations an partnerships; these, thus, reach beyond “pure” biosecurity concerns, and touch on aspects of both other pillars of bioengagement. It is, therefore, worth highlighting the distinction between the deliverable of the funded project, which is the development of the Policy and the Bill and its submission to Parliament, versus the projected impact of implementation of the Bill once passed into law. Figure 3 examines the sequential impact of the development of the Policy and Bill (solid boxes) versus those areas that will also be addressed once the Bill is implemented (transparent boxes).

**Table 2:** **Characterization of Uganda Biosecurity Policy stated outcomes within the defined pillars of bioengagement programs**.

Uganda Biosecurity Policy stated outcomes (20)	Bioengagement pillar
Ensure emergency preparedness, at the field, community, and health facility levels	Disease surveillance, detection, diagnosis, and control
Facilitate early detection of and response to emerging disease threats	Disease surveillance, detection, diagnosis, and control
Ensure integrated response to threats and rationalization of controls	N/A
Put in place the containment principles, technologies, and practices that are implemented, to prevent the unintentional exposure to pathogens and toxins, or their accidental or intentional release	Biosafety and biosecurity capacity building
Reduce the risk of biothreats by guiding the development of safety and security standards that are consistent with international guidelines and requirements	Biosafety and biosecurity capacity building
Create opportunities for capacity building to generate a critical mass of scientific and technological expertise in biorisk management	Biosafety and biosecurity capacity building
Promote collaborations, partnerships, and linkages at national, regional, and international levels to provide inclusive, effective, affordable, and practical solutions to pressing local and international concerns	Cooperative biological research

Overall, the focus of the Policy and Bill development project align very closely with GHSA Prevent 3, with respect to creating a national framework for biosafety and biosecurity (Figure 3). There is less alignment with IHR; however, actual implementation of the Policy and Bill will address IHR core capacities, such as Core Capacity 1 (National legislation, policy, and financing) and Core Capacity 8 (Laboratories) and therefore Policy and Bill development can be seen to be acting in support of IHR.

#### Kenya Zoonotic Disease Unit

The Kenya ZDU was launched in August 2012 as a joint initiative between the then Ministry of Public Health and Sanitation [now the Ministry of Health (MoH)] and the then Ministry of Livestock Development [now the Ministry of Agriculture, Livestock, and Fisheries (MALF)] (22). Support for the effort, including construction of the building housing the unit and the development of the strategic plan, were provided by CBEP and the US BEP (23), with technical assistance from GIS, a US BEP grantee (24). Both programs continue to support ZDU activities, including the drafting of guidelines for priority zoonotic diseases and a regional One Health conference hosted by the ZDU (24, 25).

The goals for the ZDU, as described in the 2012–2017 Strategic Plan, can be summarized as improving surveillance and control of zoonotic diseases; establishing partnerships related to One Health; and conducting research on zoonotic pathogens (Table 3). These align closely with the disease surveillance and cooperative research pillars of bioengagement.

**Table 3:** **Characterization of the goals of the Republic of Kenya Zoonotic Disease Unit (per the 2012–2017 Strategic Plan) within the defined pillars of bioengagement programs**.

Kenya Zoonotic Disease Unit goals (2012–2017)	Bioengagement pillar
To strengthen surveillance, prevention, and control of zoonoses in both humans and animals	Disease surveillance, detection, diagnosis, and control
To establish structures and partnerships that promotes One Health approaches	Disease surveillance, detection, diagnosis, and control
To conduct applied research at the human–animal–ecosystem interface in order to better understand the mechanism of maintenance and transmission of zoonotic pathogens	Cooperative biological research

**Figure 4:**
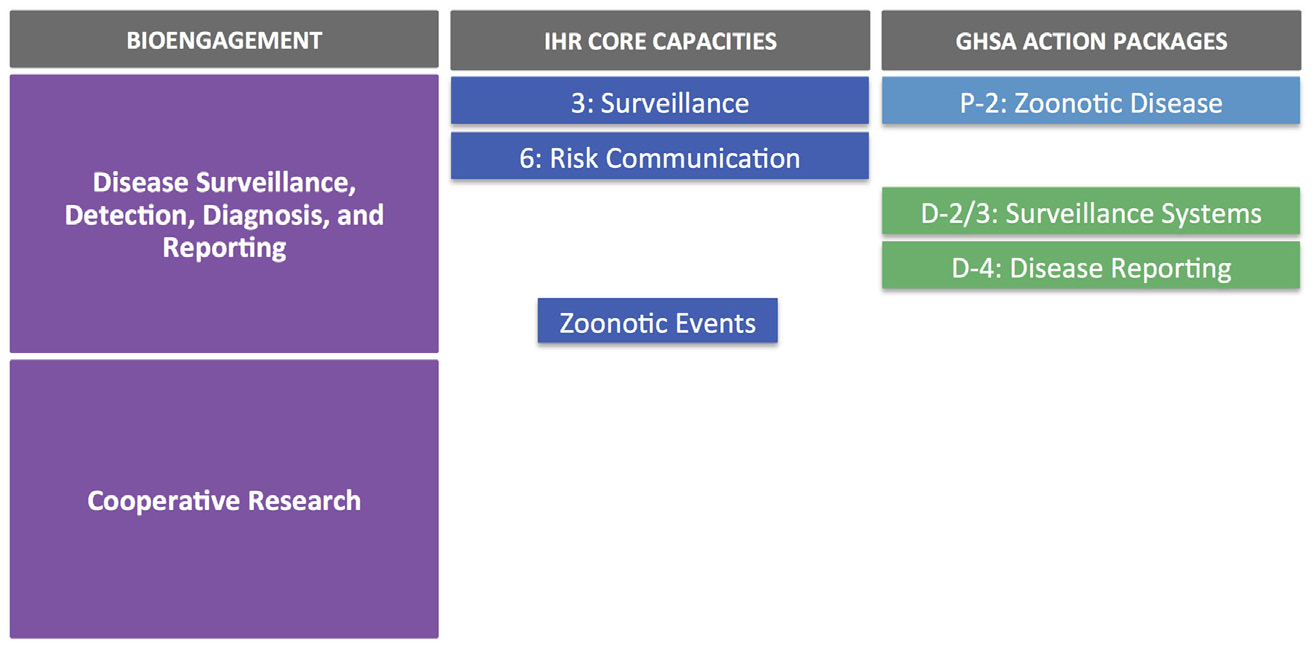
**Republic of Kenya Zoonotic Disease Unit case study**.

In terms of alignment with IHR and GHSA objectives, the main overlaps are with core capacities/action packages associated with surveillance, reporting, and zoonotic diseases (Figure 4). It is also worth noting that the targets and measures outlined for surveillance (Detect 2/3) under GHSA do not specifically cover zoonotic diseases; suggested syndromes are provided within the text of the action package, but the choice should be based on country priorities, therefore providing an opportunity to include priority zoonotic syndromes. As seen earlier, the cooperative biological research elements of the ZDU goals do not directly correspond to any IHR core capacity indicator or GHSA action package.

#### Iraq Science Fellowship Program

The ISFP was founded in 2008 as a mechanism for creating opportunities for Iraqi scientists to further their careers and create new collaborative opportunities by spending 3–6 months at a U.S. institution while conducting a specific research project. US CTR programs and the UK BEP have both supported biological ISFP fellows in past years (26), and in 2014, CBEP also launched a biologist-specific fellowship program [known as the Iraq Biosciences Fellowship Program (IBFP)] supporting scientists from the Iraq MoH, Ministry of Agriculture, and Ministry of Science and Technology (26).

The stated objectives of ISFP and IBFP relate entirely to creating research networks, building scientific capacity, and providing the fellows with skills to advance their research careers, all of which fall under bioengagement’s cooperative biological research pillar, and particularly the focus areas related to scientist engagement (Table 4). However, an examination of the scientific disciplines given selection preference by IBFP (27) indicate that beyond these stated objectives, there may in fact be significant additional overlap with bioengagement priorities. For example, a scientist selected to work on biosecurity policy issues at a US institution would not only constitute cooperative research but also biosecurity capacity building; likewise a fellow working on novel viral diagnostic methods would also be addressing biosurveillance objectives while part of the collaborative research effort.

**Table 4:** **Characterization of the goals and expected outcomes of the Iraq Science Fellowship Program within the defined pillars of bioengagement programs**.

Iraq Science Fellowship Program goals and outcomes (26, 40)	Bioengagement pillar
Enrich their scientific knowledge	Cooperative biological research
Develop valuable skills to promote Iraq’s scientific community	Cooperative biological research
Learn new methods and expertise to improve research capabilities	Cooperative biological research
Opportunity to increase Iraq’s scientific capacity	Cooperative biological research

**Figure 5:**
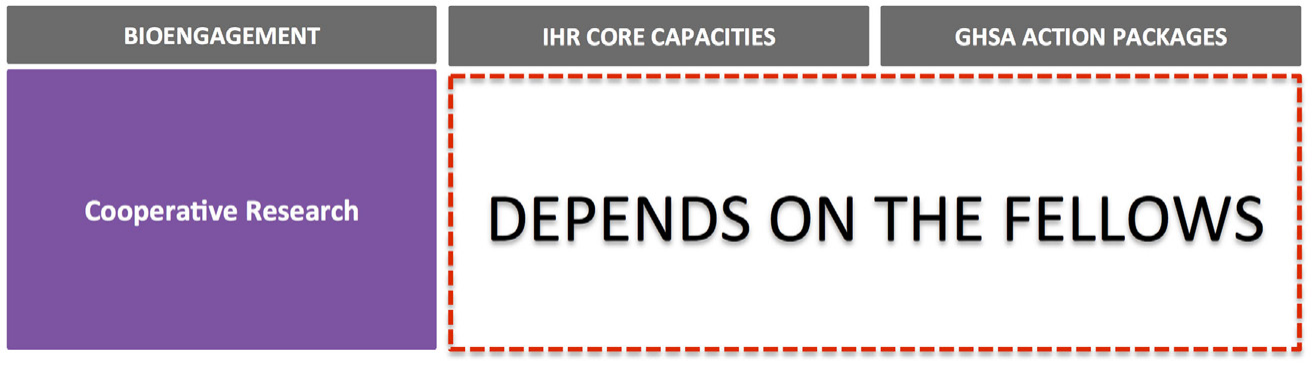
**Iraq Science Fellowship Program and Iraq Biosciences Fellowship Program case study**. Note that without greater detail on the research projects supported through these fellowships, it is difficult to determine the elements of IHR or GHSA that might be advanced through the projects; IHR and GHSA otherwise do not contain elements that align directly with collaborative research aims or scientist engagement.

Taken at face value, given that neither IHR nor GHSA explicitly contain indicators or measures related to cooperative research or scientist engagement, there is little if any overlap between ISFP/IBFP and these health security frameworks (Figure 5). However, the specific research projects conducted by the researchers participating in the fellowship programs may themselves relate back to biosafety, biosecurity, or disease surveillance efforts, which in turn could have bearing on IHR or GHSA implementation. To our knowledge, lists of ISFP and IBFP fellowship projects are not published online or available through open sources, so we were unable to determine if additional areas of alignment exist between ISFP/IBFP and IHR or GHSA at the research project level.

## Discussion

This work describes the alignment of cooperative bioengagement programmatic elements with IHR core capacities and GHSA action packages, as described through mapping of program priorities and qualitative examination of three case studies. Overall, IHR and GHSA represent opportunities to bioengagement programs that may be seeking leverage points around which to form new partnerships, and also to the implementation community, who may be able to better tailor projects and receive funding from bioengagement programs to support existing health security activities that advance IHR and GHSA compliance. These areas, notably aspects of biosafety and biosecurity capacity building and biosurveillance, could also provide an opportunity for bioengagement programs to develop evaluation measures and metrics that meet partner country targets with respect to IHR and GHSA.

However, we also observed clear gaps in the extent of overlap between some areas of bioengagement efforts and IHR/GHSA priorities. Within the biosecurity pillar in particular, there remain differences related to definitions of key terms, ideal outcomes, and the precedence placed on national-level legislative and regulatory frameworks as a mechanism to achieve sustainable implementation. For example, while IHR’s biosafety and biosecurity-related attributes are focused primarily at the level of diagnostic laboratories, the emphasis of GHSA’s Prevent 3 action package is on the development of national-level regulatory or legislative frameworks, thus targeting a different level of decision-makers and stakeholders; the Uganda Biosecurity Policy and Bill project, which we selected as our biosecurity case study, fit more closely with this latter definition. Similarly, within the veterinary sector, “biosecurity” usually does not refer to preventing unauthorized access to pathogens, but rather corresponds to the suite of control measures applied to protect against and prevent the spread of disease (28). In addition, words, such as “security” and “biosecurity,” may be associated with cultural and social sensitivities, which may differ significantly between partner countries and regions, and thus need to be taken into account when describing project outcomes and establishing new partnerships. The Kenya ZDU case study highlighted that although bioengagement programs have a strong interest in zoonotic diseases, it may prove more challenging to link zoonotic disease-focused bioengagement projects with IHR or GHSA-driven targets and measures.

The cooperative biological engagement pillar did not have clear areas of alignment with any of the IHR core capacities or GHSA action packages. There is no mention of networking between researchers or scientists, or the basic research and development underpinnings of health security in IHR or GHSA. However, when examining the case studies, it was clear that projects funded under different pillars that explicitly address IHR and GHSA objectives can also have a clearly defined research component; likewise, “pure” cooperative research projects, if focused on subject areas that related to disease surveillance, control, or biorisk management, may also result in improved IHR or GHSA compliance in the long-term. One solution to this apparent paradox could be to reconsider the three pillars of bioengagement, and rather than consider cooperative research as an end in itself, recognize it as a means for advancing sustainable capacity building for biosafety/biosecurity and biosurveillance activities. Another option could be to categorize programmatic efforts based on the target audience: i.e., national-level initiatives, facility-level initiatives, and science/knowledge-based initiatives. There is precedence for both such approaches (7, 8), and so future academic analyses of the overlaps between bioengagement and other frameworks may want to examine the impact of these alternative characterizations. Such an approach might also address the observation from the Uganda Biosecurity Bill case study that there can be a distinction between the direct outcomes of the funded project (in this case, a Policy and piece of legislation) and future projected impacts (implementation of the legislation). Adapting the way projects are characterized within bioengagement programs might provide a more explicit means for acknowledging the broad benefits that can sometimes accrue from even narrowly focused projects.

The case studies revealed several other points for further consideration. When researching case studies, we found little information on specific bioengagement-funded projects in the public domain, let alone on project outcomes, which significantly limited the extent to which we could analyze alignment with IHR/GHSA. To our knowledge, there are no universally accepted metrics consistently used across bioengagement programs; those that have been developed for specific programs, such as RAND’s effort related to DTRA’s CBEP (7), have not been used publicly to evaluate project success, but rather may be kept for internal use within the program. While it is possible to conduct convincing meta-analyses without shared metrics, the lack of outcome-focused data in the public domain at all related to these programs limits opportunities for such analysis. Implementers can moreover have legal restrictions on the extent to which they can publish project-related information (for example they may be constrained by Non-Disclosure Agreements), or may feel obliged not to publicize project details if they feel it could jeopardize future funding. Moreover, some implementers, particularly those with a public health mandate, may not be comfortable advertising their funding as coming from a bioengagement source due to the security connotations. Overall, these factors make it very challenging for independent, objective analysis of bioengagement programs and the extent to which they are successful at promoting compliance with health security frameworks.

**Figure 6:**
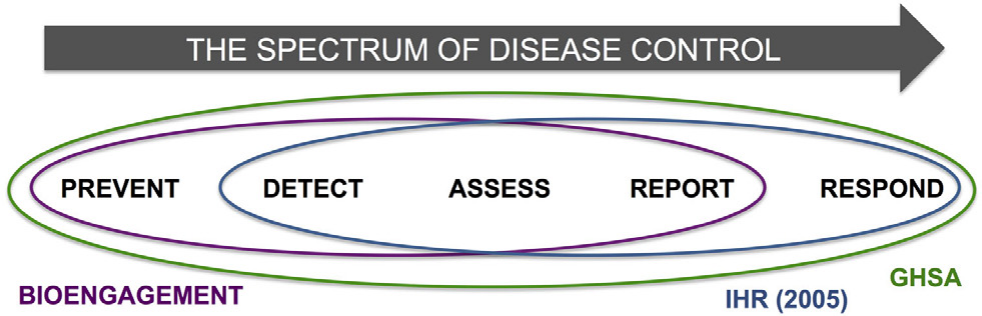
**Schematic demonstration of the spectrum of disease control, incorporating the steps of “Prevent,” “Detect,” “Assess,” “Report,” and “Respond,” and highlighting the areas of each encapsulated by bioengagement, IHR, and GHSA priorities and efforts**.

A final observation, which was largely beyond the scope of this paper but should be examined in more detail in further analytical efforts, was to note the aspects of IHR and GHSA that might be overlooked if bioengagement programs were the only groups working in a particular country or region. Thinking across the spectrum of disease control, it is notable that bioengagement programs focus primarily on prevention, detection, assessment, and reporting of disease events, rather than response; IHR and GHSA, in contrast, place a high priority on response capabilities, through core capacity 4 (Response) and action package Respond 1–3, respectively (Figure 6). While bioengagement programs have made significant investments in building emergency operations capacities in several countries [examples include Jordan (29), Vietnam (30), and Uganda (31)], these have been framed exclusively as “preparedness” efforts, and particularly among the US-funded bioengagement programs, funding is generally not available to support activities that are solely response-oriented, given their non-proliferation and prevention mandate (32). This suggests that countries seeking to form partnerships to develop their capacities to respond to disease threats may need to look outside the bioengagement donor community, or describe their needs carefully to emphasize the preparedness elements.

This descriptive exercise provided an opportunity to acknowledge the diversity of programmatic efforts throughout different international bioengagement programs, and also recognize the potential for greater alignment with parallel efforts that exist solely in the global health sphere, including those which have yet to become significant stakeholders in the on-going health security dialog. Major players in global health, for example, “traditional” vertical disease control programs, such as the President’s Emergency Plan for AIDS Relief (PEPFAR), and even non-communicable disease control efforts, are realizing their role in meeting some aspects of IHR implementation, for example in building laboratory capacity (33). Some of these efforts may moreover directly align with bioengagement priorities, even if the motivation behind the project differs. The opportunity to re-examine the donor landscape with respect to the full spectrum of disease and biological threats may, thus, provide opportunities for greater coordination between sectors. This, in turn, could have a positive impact not only on project outcomes, but also for in-country perceptions of bioengagement programs, leading in turn to deeper, more sustainable relationships with partner countries.

## Author Contributions

CS and ES conceived the concept; CS, ES, SK, JF, and RK conducted the research; CS drafted the manuscript, with technical input from ES, SK, JF, and RK; all authors reviewed and approved the final version.

## Conflict of Interest Statement

Rebecca Katz is Principal Investigator (PI) on one project funded by DTRA CBEP. Julie E. Fischer is PI on two US BEP-funded grants. Erin M. Sorrell, Sarah Kornblet, Julie E. Fischer, and Rebecca Katz receive salary support from DTRA CBEP and US BEP projects; Claire J. Standley receives salary support from the DTRA CBEP project only. Erin M. Sorrell and Claire J. Standley previously served as contractors within US BEP through February 2014 and August 2014, respectively.
