# Supplementary material for: Implementation of the International Health Regulations (2005) Through Cooperative Bioengagement
Source: Front Public Health. 2015 Oct 13;3:231. doi: 10.3389/fpubh.2015.00231 (PMC4602103; doi:10.3389/fpubh.2015.00231)
Supplement: Supplementary file 1 [file Data_Sheet_1.DOCX]

**Supplemental file 1:**

Key words used in search for cooperative biological engagement projects with sufficient available open source detail for alignment against bioengagement pillars as well as IHR and GHSA objectives.

The below table summarizes the primary findings of the keyword search. None of the sources identified contained sufficient detail for our analysis, so we proceeded instead with a convenience sample of three projects identified through the authors’ own knowledge of the field and for which sufficient information on objectives and goals was available publicly.

| Source | Summary description | Website |
| --- | --- | --- |
| AAAS | Project report on biosecurity engagement in the Middle East and North Africa (MENA) funded by BEP. The report does not propose or measure capacity building outcomes. | http://www.aaas.org/sites/default/files/reports/AAAS%20MENA%20Cooperation%20Report%20v.2%202013.pdf |
| AAAS | Funded by NPS (indirectly a CTR-funded project), this report outlines a pathway for future implementation of the findings from the aforementioned BEP-funded AAAS report on MENA. | http://www.nps.edu/Academics/Centers/CCC/PASCC/Publications/2013/Roadmap%20-%20AAAS%20Future%20MENA%20Bioengagement%20123013.pdf |
| AECOM | Short description of CBEP support work in Kazakhstan. | <http://www.aecom.com/What+We+Do/Government/Logistics,+Operations+and+Maintenance/_>projectsList/Cooperative+Biological+Engagement+Program+(CBEP) |
| Armscontrol.org | Analysis of Global Partnership efforts as a whole with no programmatic details. | https://www.armscontrol.org/act/2013_04/The-Global-Partnership-on-WMD-A-Work-in-Progress |
| APEIR | Summary of kick-off meeting with ASEAN and GPP for health cooperation on emerging infectious diseases. | http://www.apeiresearch.net/new/other.php?content=events&subcontent=eventinfo&eid=20 |
| USDA ARS | Summary description of the overall cooperative agreement between State BEP and the USDA ARS. | http://www.ars.usda.gov/research/projects/projects.htm?accn_no=420265 |
| BEP website | Generic BEP website (not hosted on State Department server) with overview of programmatic areas of focus and grant opportunities. No specific projects listed. | http://bepstate.net/ |
| BEP presentation | Presents detail related to programmatic accomplishments circa 2007; does not mention specific objectives for the projects, or even who implemented them. | http://www.biosecurity.sandia.gov/ibtr/subpages/pastConf/20062007/cairo/BEPPresentation-2.pdf |
| Black & Veatch | Anecdotal narrative of project success in Armenia supported by CBEP | http://bv.com/Projects/dtra-armenia-cooperative-biological-engagement-program |
| BMC Public Health | Academic paper (Katz et al, 2010) describing U.S. government support for global biosurveillance activities. CBEP and BEP are mentioned, but no specific projects are described in detail. | http://www.biomedcentral.com/1471-2458/10/S1/S13 |
| Biological Weapons and Toxins Convention | U.S. government one-pagers providing programmatic summaries for BEP and CBEP, among other programs. | http://geneva.usmission.gov/wp-content/uploads/2012/12/article-x-one-pagers-1128121.pdf |
| CBEP | Outlines strategic plan for addressing biological threat reduction through research. | http://biogirl757.weebly.com/uploads/3/9/6/8/39680816/cbep_research_strategy.pdf |
| FAS | Issue brief on placing biosecurity engagement within the umbrella of global health security. | http://fas.org/wp-content/uploads/2014/05/bio-engagement-final-may-2014.pdf |
| Frontiers | The call for manuscripts from Frontiers for this issue came up in our keyword search. | http://journal.frontiersin.org/researchtopic/3482/biological-engagement-programs-reducing-threats-and-strengthening-global-health-security-through-sci |
| Office of Cooperative Threat Reduction | Official State Department site for the Office of Cooperative Threat Reduction, with a brief overview of BEP. | http://www.state.gov/t/isn/offices/c55411.htm |
| Office of Cooperative Threat Reduction FY 2009 report | Mentions broad program focus areas and lists a selection of partner countries and regions. | http://www.state.gov/documents/organization/159277.pdf |
| URC | One paragraph overview of work in Iraq and Afghanistan supported by CBEP. | http://www.urc-chs.com/project?ProjectID=295 |
| U. Wash I-TECH | One sentence description of CBEP-funded disease surveillance and laboratory training project in Tanzania. | http://globalhealth.washington.edu/node/9730 |
